# Examining the psychometric properties of a split version of the EQ-5D-5L anxiety/depression dimension in patients with anxiety and/or depression

**DOI:** 10.1007/s11136-023-03372-7

**Published:** 2023-02-21

**Authors:** Yared Belete Belay, Cathrine Mihalopoulos, Yong Yi Lee, Brendan Mulhern, Lidia Engel

**Affiliations:** 1 School of Public Health and Preventive Medicine, Monash University, Level 4, 553 St Kilda Road, Melbourne, VIC 3004 Australia; 2 School of Pharmacy, Mekelle University, Mekelle, Ethiopia; 3 School of Public Health, The University of Queensland, Brisbane, Australia; 4 Queensland Centre for Mental Health Research, Brisbane, Australia; 5 Centre for Health Economics Research and Evaluation, University of Technology Sydney, Sydney, Australia

**Keywords:** Anxiety, Depression, EQ-5D, Composite domain, Outcome measurement

## Abstract

**Purpose:**

This study explored differences in self-reported responses and the psychometric performance of the composite EQ-5D-5L anxiety/depression (A/D) dimension compared with a split version of the dimension where ‘anxiety’ and ‘depression’ are measured separately.

**Methods:**

People with anxiety and/or depression who visited the Amanuel Mental Specialized Hospital in Ethiopia completed the standard EQ-5D-5L with the added subdimensions. Correlation analysis was used to examine convergent validity with validated measures of depression (PHQ-9) and anxiety (GAD-7), while ANOVA was used to assess known-groups’ validity. Agreement between ratings for composite and split dimensions was compared using percent agreement and Cohen’s Kappa, while the proportion of ‘no problems’ reports was compared using the chi-square test. Discriminatory power analysis was undertaken using the Shannon index (H’) and Shannon Evenness index (J’). Open-ended questions explored participants' preferences.

**Results:**

Of the 462 respondents, 30.5% reported no problems with the composite A/D, while 13.2% reported no problems on both subdimensions. Agreement between ratings for composite and split dimensions was highest for respondents with comorbid anxiety and depression. The depression subdimension had higher correlation with PHQ-9 (*r* = 0.53) and GAD-7 (*r* = 0.33) than the composite A/D dimension (*r* = 0.36 and *r* = 0.28, respectively). The split subdimensions and composite A/D could adequately differentiate respondents based on their severity of anxiety or depression. Slightly better informativity was observed in EQ-4D-5L + anxiety (*H’* = 5.4; *J’* = 0.47) and EQ-4D-5L + depression (*H’* = 5.31; *J’* = 0.46) than EQ-5D-5L (*H’* = 5.19; *J’* = 0.45).

**Conclusions:**

Adopting two subdimensions within the EQ-5D-5L tool appears to perform slightly better than the standard EQ-5D-5L.

**Supplementary Information:**

The online version contains supplementary material available at 10.1007/s11136-023-03372-7.

## Introduction

The EQ-5D is a generic multi-attribute utility tool widely used to measure health-related quality of life (HRQoL) and to guide resource allocation decisions in healthcare [1, 2]. It has five domains: mobility (MO), self-care (SC), usual activities (UA), pain/discomfort (P/D) and anxiety/depression (A/D). Each dimension is measured on a three-level (EQ-5D-3L) or five-level (EQ-5D-5L) response scale [3]. Despite being the most commonly used measure of health status, there is ambiguity in the health dimensions of the EQ-5D, particularly related to the two composite dimensions, P/D and A/D, that combine two health constructs within a single dimension [1, 4]. Combining two dimensions in one question may create response challenges. For instance, if a patient feels slightly depressed and moderately anxious, it may be challenging to obtain an appropriate report on the composite dimension of the EQ-5D. To date, only a few studies have explored the use and interpretation of the composite dimensions of the EQ-5D in self-report and valuation [4–8]. The results suggest that respondents mainly use the P/D dimension to report pain, while the A/D dimension is largely used to report problems on the component that is considered most severe [7].

A recent study explored the measurement properties of the EQ-5D-5L P/D by drawing comparisons to pain or discomfort items included in the EQ Health and Wellbeing (EQ-HWB) measure [6, 9]. It demonstrated that the EQ-5D-5L P/D dimension captures aspects of pain more than aspects of discomfort, which could be driven by the absence of descriptors for discomfort (e.g. itching) or because pain is mentioned first in the composite dimension. Without explicit descriptors, respondents may interpret discomfort as a mild form of pain and not associate other forms of discomfort [6]. In another study, respondents described pain, such as headaches or musculoskeletal pain, as a kind of discomfort [4]. However, the same may not hold for the A/D of the EQ-5D-5L, where evidence has shown that people consider anxiety and depression as two distinct and independent constructs [5]. This requires further investigation.

While previous studies have consistently demonstrated that people respond differently to the composite dimensions compared with its split versions [4–8], there is a lack of studies that examined the psychometric properties of these by drawing comparisons to widely validated and accepted disease-specific tools. Further research is required to explore the validity of the composite versus split version of the A/D dimension of the EQ-5D-5L in comparison with other valid tools, such as the Generalised Anxiety Disorder-7 (GAD-7) scales for measuring the severity of anxiety [10] or the Patient Health Questionnaire-9 (PHQ-9) scales for measuring the severity of depression [11]. Furthermore, the findings that compared the split versus composite versions in earlier studies were mainly generated from online panel members with/without health conditions and there is currently a lack of evidence from clinical settings. Previous methodological studies have also been undertaken in high-income countries using the English version of the EQ-5D-5L. The performance of the composite dimensions of the EQ-5D-5L in low-income countries and languages other than English is lacking. This study aimed to explore differences in self-reported responses to the composite EQ-5D-5L A/D dimension and split subdimensions; and to evaluate the psychometric performance in respondents with anxiety and/or depression in a low-income hospital setting.

## Methods

### Study setting and recruitment

This study used a cross-sectional study design to recruit participants from Amanuel Mental Specialised Hospital (AMSH), the only specialised psychiatric hospital serving people from all regions of Ethiopia. Respondents with anxiety and/or depression who visited the hospital for outpatient services were eligible to participate in this study. After completing their outpatient visit, research nurses who had access to patients' records (patient case notes) invited eligible patients to an interview, which was done in a separate room where clinicians or other hospital staff were not involved. The sample size for the current study was determined using a single proportion formula [12]. A proportional number of participants (around one-third of each) with anxiety (149), depression (159) and with both anxiety and depression (154) were recruited on a consecutive basis until the target sample was reached.

### Data collection

Five research nurses collected data between June and October 2021. The interviewer-administered approach was used to collect data, given that self-report is associated with some challenges due to low literacy levels among the Ethiopian population. The Amharic version of the EQ-5D-5L was used, which was translated as part of the Ethiopian valuation study using the standardized approach recommended by the EuroQol group [13, 14]. The EQ-5D-5L has been previously used in other studies in Ethiopia [15–17]. Validated and commonly used tools to measure anxiety and depression, the GAD-7 and the PHQ-9 scales, were used to assess the severity of anxiety and depression. The severity of depression was classified based on PHQ-9 scores (1 to 4 points = minimal, 5 to 9 points = mild, 10 to 14 points = moderate, 15 to 19 points = moderately severe and 20 to 27 points = severe) [11]. The GAD-7 scores calculated from seven questions were used to classify the level of anxiety (0 to 4 minimal, 5 to 9 mild, 10 to 14 moderate and 15 to 21 severe) [10]. During the data collection, respondents were asked to complete the EQ-5D-5L with amended split version of the anxiety and depression subdimensions in a random order after the main descriptive system, followed by the PHQ-9 and the GAD-7. Two survey versions were used: one with standard EQ-5D-5L questions followed by split anxiety and depression subdimensions in sequential order and a second version where the split anxiety and depression subdimensions were placed between P/D and A/D questions of the standard EQ-5D-5L. After completing the EQ-5D-5L questions, participants were asked to select whether they preferred the composite EQ-5D-5L A/D question or the split questions. They were then asked an open-ended question to describe the reasons for their preferences. The data collection tool also included questions related to respondents’ socio-demographic characteristics and clinical characteristics. The type of diagnosis (including types of anxiety and depression) was based on the medical diagnosis by clinicians (psychiatrists) at the AMSH, while the severity of anxiety or depression was based on the self-report from the respondents. In all questions, except the clinical characteristics, Amharic, the official working language of the country, was used to ensure comprehensibility of the questions by the patients.

### Analyses

The data were entered into Excel and then imported into STATA version 14, where most statistical analyses were carried out. The informativity analysis were done in Excel. A sankey diagram was generated using R-Studio to show the relationship between EQ-5D-5L response levels and the severity of anxiety or depression. Descriptive statistics were used to describe the study population by demographic and clinical characteristics, including frequency and percentage. The proportions of ‘no problems’ reports were compared using the chi-square test. We have used the more common convention of a p value of less than 0.05 to denote statistical significance. Agreement between rating for composite and split dimension was compared using percent agreement and Cohen’s Kappa (*k*) statistics, with scores interpreted as k: ≤ 0 = poor, 0.01–0.20 = slight, 0.21–0.40 = fair, 0.41–0.60 = moderate, 0.61–0.80 = substantial and 0.81–1 = almost perfect [18]. Convergent validity was assessed by examining the correlation (Spearman’s rank correlation coefficients) between all EQ-5D-5L dimensions, the split dimensions and measures of anxiety severity and depression severity (i.e. GAD-7 and PHQ-9). Correlation coefficient was interpreted as weak (≤ 0.4), moderate (0.4–0.7) and strong (> 0.7) [19]. To examine the known-group validity, one-way ANOVA was used to compare the mean scores of the EQ-5D-5L A/D and the split dimensions. Scheffe’s post hoc comparisons and effect size (Cohen's d statistic) were used to show the difference in mean values between all possible pairs of groups. Effect sizes of less than 0.2 are considered as small, around 0.5 as moderate and greater than 0.8 as large [20]. The Shannon index (H') and Shannon Evenness index (J’) were used to demonstrate the power of informativity of the composite and split dimensions. The H’ value represents absolute information captured by possible categories.

The Shannon index (Hʹ) was defined by

$$H^{\prime} = - \sum\limits_{i = 1}^{c} {pi\log 2pi}$$

With *pi* being the proportion of respondents with one specific health profile (e.g. 11,223), and c being the total number of theoretically possible health profiles (i.e. 3125 for the EQ-5D-5L, and 15,625 for the EQ-4D-5L + anxiety + depression). If a profile is not observed, its contribution is zero to this summation. For example, when comparing the EQ-5D-5L A/D composite (five dimensions) version to the split EQ-6D-5L version (6 dimensions), we expect the H ’ of the split to be higher to reflect more discriminatory performance. The higher the H’ value, the more information is captured by the system, and maximum H' (H'max) is observed in homogenous distribution. The J’ value represents the extent to which information is evenly distributed (relative informativity) across the categories and estimated by dividing H’ by H’max [21]. The free text describing the reasons for patient preferences towards composite versus split questions was analysed using content analysis, which is a qualitative data analysis method that is used to identify common patterns and themes within the data [22]. One author (YB) read and re-read the text comments, generated codes, and organised those into categories.

## Results

Data from all (462) participants were included in the analysis, with no missing responses due to the nature of the data collection process via interviews. Table 1 includes a summary of the sample characteristics. According to participants' medical records, 64.7% had a major depressive disorder (MDD) diagnosis, followed by generalised anxiety disorder (55.8%). Participants had mild to severe depression, with a slightly higher proportion (33.6%) reporting moderate depression and the remainder of respondents' scores being about evenly divided across the mild, moderately severe and severe levels. In addition, participants reported mild to severe anxiety based on GAD-7 scores, with a higher percentage reporting a mild (37.5%) and moderate (40%) severity level (Table 1).

**Table 1:** Demographic and clinical characteristics of the study population [frequency, *n* (%)]

Demographic characteristics		*n* (%)	Clinical characteristics		*n* (%)
Gender	Male	231 (50)	Diagnosis	Anxiety	149 (32.3)
	Female	231 (50)		Depression	159 (34.4)
Age (in years)	< 35	249 (53.9)		Both	154 (33.3)
	36–55	183 (39.6)	Type of depressive disorder	No depressive disorder	149 (32.3)
	> 55	30 (6.5)		Major depressive disorder	299 (64.7)
Marital status	Single	209 (45.2)		Dysthymia	3 (0.7)
	Married	174 (37.7)		Premenstrual dysphoric disorder	4 (0.9)
	Divorced	57 (12.3)		Other^c^	7 (1.5)
	Widowed	22 (4.8)	Type of anxiety disorder	No anxiety disorder	159 (34.4)
Education	No formal education	52 (11.3)		Generalized anxiety disorder	259 (56.1)
	Primary education	142 (30.7)		Social Phobia/Specific phobia	20 (4.3)
	Secondary education	136 (29.4)		Agoraphobia	3 (0.7)
	TVT /diploma^a^	69 (14.9)		Anxiety disorder due to another medical condition	7 (1.5)
	Degree and above	63 (13.6)		Other^d^	14 (3)
Occupation	Employed	227 (49.1)	Duration of illness (in years)	< 5	343 (74.2)
	Pensioner	14 (3)		5–10	69 (15)
	Student	81 (17.5)		> 10	50 (10.8)
	Looking after home or family	65 (14.1)	Co-morbid medical diagnosis	No comorbid diagnosis	391 (84.6)
	Other^b^	75 (16.2)		DM	26 (5.6)
Income	Not enough	308 (66.7)		HTN^e^	25 (5.4)
	Just enough	129 (27.9)		Other^f^	20 (4.3)
	More than enough	25 (5.4)	Medications for mental illness	Antidepressant	242 (52.4)
Smoking habit	Daily	32 (6.9)		Antipsychotic	26 (5.6)
	Less than daily	62 (13.4)		Sedative-hypnotic	6 (1.3)
	Not at all	368 (79.7)		Mood stabilizer	5 (1.1)
Alcohol consumption habit	Never	283 (61.4)		Multiple	181 (39.2)
	Once a month	79 (17.1)		No medications	2 (0.4)
	2–4 times a month	54 (11.7)			
	2–3 times a week	36 (7.8)	Anxiety severity (based on GAD-7 scores)	Minimal (0–4)	–
	4 or more times a week	9 (2)		Mild (5–9)	173 (37.45)
				Moderate (10–14)	185 (40.04)
				Severe (15–21)	104 (22.51)
			Depression severity (based on PHQ-9 scores)	Minimal (0–4)	–
				Mild (5–9)	109 (23.59)
				Moderate (10–14)	155 (33.55)
				Moderately severe (15–19)	97 (21)
				Severe (20–27)	101(21.86)

^a^TVT-technical and vocational training

^b^Unemployed, seasonal

^c^Substance-induced depressive disorder

^d^Post-traumatic stress disorder

^e^HNT-hypertension

^f^Pneumonia, malaria, febrile illness, asthma, breast cancer

### EQ-5D-5L response distribution

Most participants reported that they had no problems on MO (78.8%), SC (73.6%) and UA (57.4%). Less than half of the respondents reported they had no problems on the composite P/D (43.9%) or A/D (30.5%). The distribution of responses across the EQ-5D-5L and the split anxiety and depression subdimensions is presented in Appendix 2 in the Supplementary File. In the split version, 26% and 23.8% reported no problems on the anxiety and depression subdimensions, respectively, while 13.2% reported no problems on both the anxiety and depression subdimensions. The proportion of no problem reports on the composite and split questions was significantly different [*χ2*(1, *N* = 924) = 40.55, *p* < 0.001]. Cross-tabulation analyses by clinical diagnoses in Table 2 showed that the proportion reporting no problems on the composite A/D dimension was 30.9% in patients with anxiety, 52.2% in patients with depression and 7.8% in patients with both anxiety and depression. Conversely, the proportion of no problems on both anxiety and depression subdimension was 12.8% in respondents with anxiety, 24.5% in respondents with depression and 1.9% in respondents with both anxiety and depression. These proportions were significantly different for respondents with anxiety [*χ2*(1, *N* = 298) = 14.34, *p* < 0.001], depression [*χ2*(1, *N* = 318) = 25.75, *p* < 0.001] or for respondents with both anxiety and depression [*χ2*(1, *N* = 308) = 5.68, *p* = 0.017]. The details of the chi-square test are presented in Appendix 3 in the Supplementary File.

**Table 2:** Distribution of responses of the anxiety and depression subdimensions by EQ-5D-5L A/D levels-frequency with column percentage *n* (%)

Respondents based on diagnosis	Anxiety/depression (Split)		EQ-5D-5L A/D						
			No	Slight	Moderate	Severe	Extreme	% Agreement	*k*
Total sample	Anxiety subdimension	No	*88 (62.41)*	25 (13.09)	5 (5.38)	1 (3.33)	1 (14.29)	59.1	0.42
		Slight	36 (25.53)	*116 (60.73)*	28 (30.11)	1(3.33)	0		
		Moderate	14(9.93)	38 (19.9)	*51 (54.84)*	12 (40)	3(42.86)		
		Severe	2 (1.42)	8 (4.19)	9 (9.68)	*15 (50)*	0		
		Extreme	1 (0.71)	4 (2.09)	0	1 (3.33)	*3 (42.86)*		
	Depression subdimension	No	*86 (60.99)*	16 (8.38)	7 (7.53)	1 (3.33)	0	64.1	0.49
		Slight	43 (30.5)	*130 (68.06)*	22 (23.66)	1 (3.33)	1 (14.29)		
		Moderate	7 (4.96)	35 (18.32)	*55 (59.14)*	5 (16.67)	0		
		Severe	4 (2.84)	10 (5.24)	9 (9.68)	*22 (73.33)*	3 (42.86)		
		Extreme	1 (0.71)	0	0	1 (3.33)	*3 (42.86)*		
Respondents with anxiety	Anxiety subdimension	No	*24 (52.17)*	11 (15.28)	1 (4.17)	0	0	47	0.24
		Slight	15 (32.61)	*33 (45.83)*	7 (29.17)	0	0		
		Moderate	6 (13.04)	18 (25)	*11 (45.83)*	4 (66.67)	1 (100)		
		Severe	1 (2.17)	6 (8.33)	5 (20.83)	*2 (33.33)*	0		
		Extreme	0	4 (5.56)	0	0	*0*		
	Depression subdimension	No	*31 (67.4)*	10 (13.89)	4 (16.67)	0	0	67.1	0.48
		Slight	13 (28.26)	*54 (75)*	7 (29.17)	1 (16.67)	0		
		Moderate	2 (4.34)	7 (9.72)	*13 (54.17)*	2 (33.33)	0		
		Severe	0	1 (1.39)	0	*2 (33.33)*	1 (100)		
		Extreme	0	0	0	1 (16.67)	*0*		
Respondents with depression	Anxiety subdimension	No	*59 (71.1)*	11 (23.4)	4 (25)	1 (10)	1 (33.33)	64.8	0.45
		Slight	17 (20.5)	*29 (61.7)*	5 (31.25)	0	0		
		Moderate	6 (7.2)	5 (10.64)	*7 (43.75)*	3 (30)	0		
		Severe	1 (1.2)	2 (4.26)	0	*6 (60)*	0		
		Extreme	0	0	0	0	*2 (66.67)*		
	Depression subdimension	No	*50 (60.24)*	5 (10.64)	3 (18.75	1 (10)	0	56	0.36
		Slight	25 (30.12)	*21 (44.68)*	2 (12.5)	0	1 (33.33)		
		Moderate	5 (6.02)	13 (27.66)	*7 (43.75)*	0	0		
		Severe	3 (3.61)	8 (17.02)	4 (25)	*9 (90)*	0		
		Extreme	0	0	0	0	*2 (66.67)*		
Respondents with both anxiety and depression	Anxiety subdimension	No	*5 (41.67)*	3 (4.17)	0	0	0	64.9	0.45
		Slight	4 (33.33)	*54 (75)*	16 (30.19)	1 (7.14)	0		
		Moderate	2 (16.67)	15 (20.83)	*33 (62.26)*	5 (35.71)	2(66.67)		
		Severe	0	0	4 (7.55)	*7 (50)*	0		
		Extreme	1 (8.33)	0	0	1 (7.14)	*1 (33.33)*		
	Depression subdimension	No	*5 (41.67)*	1 (1.39)	0	0	0	69.5	0.53
		Slight	5 (41.67)	*55 (76.39)*	13 (24.53)	0	0		
		Moderate	0	15 (20.83)	*35 (66.04)*	3 (21.43)	0		
		Severe	1 (8.33)	1 (1.39)	5 (9.43)	*11 (78.57)*	2		
		Extreme	1 (8.33)	0	0	0	*1*		

#### Agreement in response

Percent agreement and kappa statistics are reported in Table 2. Percent agreement of responses between the split subdimensions and the EQ-5D-5L A/D dimension ranged between 47% (anxiety subdimension in respondents with anxiety) and 69.5% (depression subdimension in respondents with both anxiety and depression). Except for those with depression, the depression subdimension had a higher percent agreement with the EQ-5D-5L A/D than the anxiety subdimension. According to the kappa statistic, there was a fair to moderate agreement. The overall agreement of anxiety and depression subdimensions with the EQ-5D-5L A/D in the total sample was moderate, with k values of 0.42 and 0.49, respectively. The depression subdimension had the highest agreement among patients with anxiety and depression (k = 0.53).

### Convergent validity assessment

In comparison to the anxiety subdimension (*r* = 0.58), the depression subdimension had a higher correlation(*r* = 0.65) with the EQ-5D-5L A/D, although the anxiety and depression subdimensions had lower correlations (*r* = 0.48) with each other. Furthermore, the depression subdimension had a higher correlation with PHQ-9 (*r* = 0.53) and GAD-7 (*r* = 0.33) than the composite EQ-5D-5L A/D dimension (*r* = 0.36 and *r* = 0.28, respectively), indicating minimally better convergent validity (Table 3).

**Table 3:** Correlation matrix (Spearman’s rank correlation coefficients) between EQ-5D-5L dimensions, the anxiety and depression subdimensions, PHQ-9 and GAD-7 score

	EQ-5D-5LMO	EQ-5D-5L SC	EQ-5D-5L UA	EQ-5D-5L P/D	EQ-5D-5L A/D	Anxiety subdimension	Depression subdimension	PHQ-9	GAD-7
EQ-5D-5L MO	1								
EQ-5D-5L SC	0.60*	1							
EQ-5D-5L UA	0.49*	0.64*	1						
EQ-5D-5L P/D	0.38*	0.49*	0.67*	1					
EQ-5D-5L A/D	0.2*	0.22*	0.38*	0.53*	1				
Anxiety subdimension	0.16	0.19*	0.26*	0.39*	0.58*	1			
Depression subdimension	0.15	0.22*	0.34*	0.49*	0.65*	0.48*	1		
PHQ-9	0.15	0.29	0.30	0.37	0.36*	0.28*	0.53*	1	
GAD-7	0.11	0.24	0.24	0.33	0.28*	0.29*	0.33*	0.64*	1

*p value < 0.0001

*MO* Mobility; *SC* Selfcare; *UA* Usual activity; *P/D* Pain/discomfort; *A/D* Anxiety/depression

### Known-group validity assessment

**Fig. 1:**
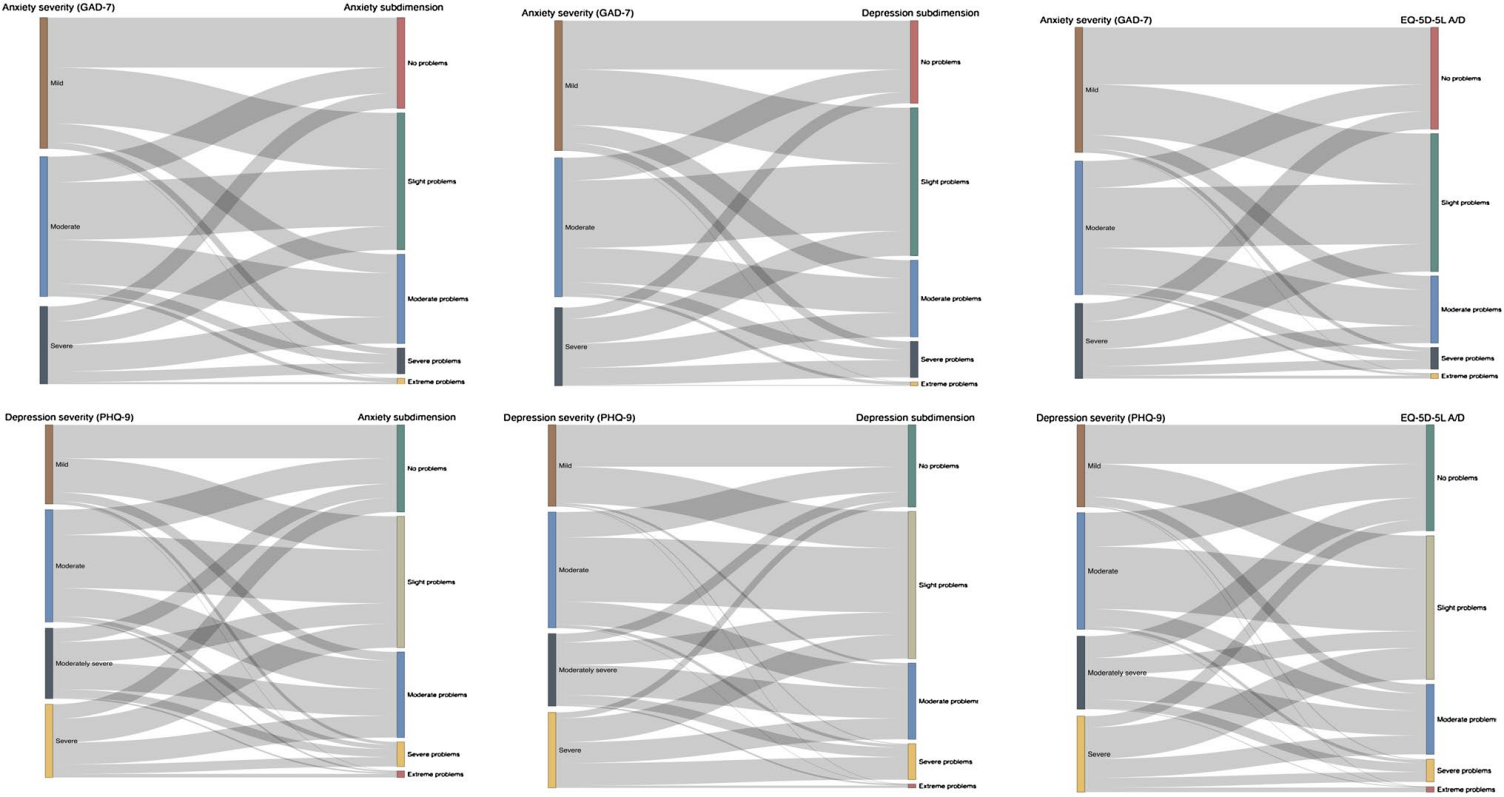
Distribution of responses towards the composite EQ-5D-5L A/D and split dimensions across the severity of anxiety and depression

The Sankey diagram in Fig. 1 shows that a greater proportion of respondents who indicated moderate anxiety or depression in symptom scores reported ‘slight problems’ on the composite and split subdimensions. In contrast, many respondents who reported mild anxiety or depression based on symptom scores selected ‘no problems’ and ‘slight problems’ on the EQ-5D’s composite and split dimensions. To further examine this relationship, a one-way ANOVA compared the mean scores by symptom severity of anxiety and depression. Composite EQ-5D-5L A/D and split subdimensions distinguished participants based on the diagnoses and illness severity (i.e. clinical diagnosis and self-reported symptom scores) with significant differences in the mean score (*p* < 0.001). Scheffe's post hoc analyses revealed that both split subdimensions and composite dimension significantly discriminated groups by anxiety severity (*p* < 0.001); however, no significant difference was reported between respondents with moderate and severe anxiety. Furthermore, the largest effect size (0.81) was reported for the depression subdimension, followed by the anxiety subdimension (0.72) and the EQ-5D-5L A/D dimension (0.71). Furthermore, the depression subdimension showed a large effect size (1.63) between depression severity groups (severe vs mild depression), followed by the EQ-5D-5L A/D (0.97) and anxiety subdimensions (0.78). Overall, both the split and composite dimensions demonstrated good known-group validity, with moderate to large effect sizes, especially between groups with mild and severe conditions (Table 4).

**Table 4:** One-way ANOVA test results

			EQ-5D-5L A/D			Anxiety subdimension			Depression subdimension		
Clinical characteristics		Sample size *n* (%)	Mean (SD)	*p* value	Pair (Scheffe *p* value; Cohen's d)	Mean (SD)	*p* value	Pair (Scheffe *p* value; Cohen's d)	Mean (SD)	*p* value	Pair (Scheffe *p* value; Cohen’s d)
Anxiety (level of severity based on GAD-7 score)	Minimal (1)	–	–	< 0.001	2vs3 (< 0.001;0.64) 2vs4 (< 0.001;0.71) 3vs4 (0.629;0.11)	–	< 0.001	2vs3 (< 0.001;0.54) 2vs4 (< 0.001;0.72) 3vs4 (0.260;0.19)	–	< 0.001	2vs3 (0.001;0.49) 2vs4 (< 0.001;0.81) 3vs4 (0.018;0.34)
	Mild (2)	173 (37.45)	1.71 (0.78)			1.86 (0.83)			1.88 (0.86)		
	Moderate (3)	185 (40.04)	2.25 (0.90)			2.34 (0.94)			2.31 (0.91)		
	Severe (4)	104 (22.51)	2.36 (1.10)			2.53 (1.07)			2.63 (1.02)		
Depression (level of severity based on PHQ-9 score)	Minimal (1)	–	–	< 0.001	2vs3 (0.148;0.36) 2vs4 (< 0.001;0.75) 2vs5 (< 0.001;0.97) 3vs4 (0.006;0.47) 3vs5 (< 0.001;0.70) 4vs5 (0.411;0.20)	-	< 0.001	2vs3 (0.014;0.46) 2vs4 (< 0.001;0.79) 2vs5 (< 0.001;0.78) 3vs4 (0.057;0.36) 3vs5 (0.027;0.37) 4vs5 (0.997;0.03)	–	< 0.001	2vs3 (< 0.001;0.74) 2vs4 (< 0.001;1.40) 2vs5 (< 0.001;1.63) 3vs4 (< 0.001;0.73) 3vs5 (< 0.001;1) 4vs5 (0.170;0.26)
	Mild (2)	109 (23.59)	1.65 (0.71)			1.76 (0.79)			1.53(0.60)		
	Moderate (3)	155 (33.55)	1.91 (0.73)			2.14 (0.86)			2.03 (0.72)		
	Moderately severe (4)	97 (21)	2.32 (1.07)			2.47 (1.00)			2.62 (0.94)		
	severe (5)	101(21.86)	2.53 (1.08)			2.50 (1.10)			2.88 (1.02)		
Types of diagnosis	Anxiety (1)	149 (32.3)	1.95 (0.83)	< 0.001	1vs2 (0.172;0.21) 1vs3 (< 0.001;0.67) 2vs3 (< 0.001;0.81)	2.30 (1.02)	< 0.001	1vs2 (< 0.001;0.50) 1vs3 (0.110;0.24) 2vs3 (< 0.001;0.81)	1.93 (0.79)	< 0.001	1vs2 (0.186;0.21) 1vs3 (< 0.001;0.84) 2vs3 (< 0.001;0.48)
	Depression (2)	159 (34.4)	1.76 (1.00)			1.81 (0.96)			2.13 (1.11)		
	Both (3)	154 (33.3)	2.51 (0.84)			2.52 (0.79)			2.60 (0.81)		

### Informativity

Adding an anxiety or depression subdimension to a modified EQ-5D-5L tool increased absolute and relative informativity, as evidenced by the increase in both Shannon indices. EQ-4D-5L + anxiety (*H’* = 5.4; *J’* = 0.47) and EQ-4D-5L + depression (*H’* = 5.31; *J’* = 0.46) result in slightly better informativity than EQ-5D-5L (*H’* = 5.19; *J’* = 0.45). Compared to standard EQ-5D-5L, adding two split dimensions instead of the composite dimension increased both Shannon indices (*H’* = 6.39; *J’* = 0.46) (Table 5).

**Table 5:** Discriminatory power of the EQ-5D-5L/ EQ-4D-5L with and without split Anxiety/depression items

		Possible health profiles	Health profiles observed (%)	Shannon index (H’)	Shannon Evenness index (J’)
Standard EQ-5D-5L A/D		3,125	3.81	5.19	0.45
Adding one extra item to the EQ-4D-5L (without anxiety/depression)	EQ-4D-5L	625	11.84	3.97	0.43
	EQ-4D-5L + Anxiety	3,125	4.13	5.4	0.47
	EQ-4D-5L + Depression	3,125	3.97	5.31	0.46
Adding two extra items to the EQ-4D-5L (without anxiety/depression)	EQ-4D-5L + anxiety + depression	15,625	1.2	6.39	0.46

### Qualitative content analysis

The content analysis summarized why respondents preferred the split anxiety and depression subdimensions over the composite EQ-5D-5L A/D. Among the 462 individuals, 163 (35.3%) preferred the composite EQ-5D-5L A/D, whereas 299 (64.7%) preferred the split subdimensions. Respondents provided more than one justification for their preference.

Based on the pattern of occurrence, the reasons were classified into three main categories that described

1. Understanding of the questions
2. Relevance to respondents ‘health condition(s); and
3. Acceptability and perceived cognitive burden of the questions.

### Understanding of the questions

Respondents who preferred the composite EQ-5D-5L A/D question stated that the question was clear (38%), easy to respond to (5%), or straightforward (3%). In addition, respondents considered the composite EQ-5D-5L A/D question to be short and better than the two subdimensions to describe (9%) or explain (3%) their health problems.

Illustrative quote

Both feelings are well represented in the combined question and are easily understandable. The question that combines anxiety and depression describes me well.

Those who preferred the two subdimensions said that they were easier to answer (5%), while 4% of them claimed better clarity and understanding as the reasons for their preference for the split questions.

Illustrative quote

"Split questions are clearer and simple; therefore, I understand them easily and they also express how I feel today."

### Relevance to respondents’ health conditions

Some respondents preferred the combined questions because of their health conditions. Participants who preferred the composite questions in this category claimed they had both conditions (anxiety and depression). The question that described both conditions in combination was easier to answer than the split subdimension (15%). This group of participants claimed to have anxiety and depression symptoms therefore they preferred the questions that blended these two feelings.

Illustrative quote.

I have both feelings, and it is better to respond in a combined format.

Those, on the other hand, who preferred the split questions felt that the two subdimensions described their health condition in a better and more accurate way (31%). Respondents stated that they felt comfortable when being asked about anxiety or depression (3%) separately. Respondents who preferred the split subdimension, generally, seemed to experience either anxiety, depression but not both, which is why they found the split versions easier to complete.

Illustrative quote

The split anxiety question tells a lot about my health and explains it very well, but depression is not my concern.

### Acceptability and perceived cognitive burden of the questions

Some participants preferred the composite or split question because of cognitive and interview burden. It was difficult for respondents to differentiate between the two conditions, and they were confused and some reported feeling anxious to respond to the split questions. Respondents claimed they were worried about answering the split questions and preferred a combined question as it avoids talking. The split question posed an additional burden of thinking long and hard about it (3%). Some respondents said that the two health conditions have something in common and cannot differentiate the two feelings; therefore, they preferred the composite question, which was easy to comprehend and respond to.

Illustrative quote

When it is decomposed, it is a thought-provoking question; it takes time."

## Discussion

The goal of this study was to gather more evidence on the EQ-5D-5L tool by comparing the psychometric performance of the composite and split versions of the EQ-5D A/D dimension in respondents with anxiety and/or depression in a low-income clinical setting.

The ‘no problems’ report on composite A/D is supposed to mean no problems on either component (anxiety or depression subdimension). Therefore, the proportion of no problems reports on composite and both subdimensions should not be significantly different. However, this study reported a significantly higher proportion of no problems on the composite A/D dimension compared to the ‘no problems’ report on both anxiety and depression subdimensions [*χ2*(1, *N* = 924) = 40.55, *p* < 0.001], meaning that problems are under-reported on the A/D dimension. This difference could be justified by how respondents interpret the composite A/D question, where some respondents who experience either anxiety or depression but not both may be less likely to report problems because of the ambiguity of the question. For instance, if respondents had moderate anxiety and no depression, they might report problems on the anxiety subdimensions but not on the composite A/D dimension. This finding is consistent with the previous study by McDonald et al. 2020 which also reported a significant difference in problem reports [7].

In our study, we also explored response patterns by the clinical diagnoses of the participants, where the proportion of respondents reporting no problems on the composite A/D and split dimensions was compared. Results showed that respondents who had either depression, anxiety or both depression and anxiety under-reported problems on the composite A/D dimension than the split subdimensions. The response patterns are consistent with those reported by McDonald et al. [7] but contradict findings from a recently published study by Rencz et al. (2022) that showed only a small difference in self-reported problems on the composite and split A/D dimensions [4]. Rencz et al. also reported a systematic order effect, with the first mentioned subdimensions (e.g. anxiety subdimension in EQ-5D-5L A/D) being more strongly correlated with the composite dimension [4]. Our study, on the other hand, found that the depression subdimension had a slightly higher correlation (*r* = 0.65) with the composite EQ-5D-5L A/D dimension than the anxiety subdimension (*r* = 0.58). The reported discrepancy between the current study and the Rencz et al. (2022) study—which recruited participants via an online survey of the general population—could be attributable to differences in respondent characteristics (general population versus those with mental health conditions). To confirm these conflicting findings, more studies using subdomain randomisation are required to assess how the order of the two subdomains influences respondents' answers [4]. Furthermore, the qualitative analysis provided additional evidence showing that some respondents preferred composite or split questions based on how well they were related to their current state of health.

The depression subdimension had a higher correlation with the EQ-5D-5L A/D dimension than the anxiety subdimension. Furthermore, the correlation between the two subdimensions was lower than their correlation with the EQ-5D-5L A/D dimension. This might indicate that the two subdimensions measure different health constructs or that respondents interpreted the composite dimension as one of the subdimensions. This supports earlier findings demonstrating inconsistency in the interpretation of the composite dimension, in which respondents view anxiety and depression subdimensions as different health constructs [5] or they used to report the subdimension out of the composite dimension for which they have more severe problems [7]. Compared to the composite EQ-5D-5L A/D dimension, the depression subdimension exhibits a stronger association with symptom scores, suggesting better convergent validity. However, it is necessary to acknowledge limitations associated with only few respondents indicating being in levels 4 and 5 for any of the items (i.e. there are not many observations at the more severe levels to support this psychometric analysis). In this context, it is important to note that although study participants were recruited from a specialised psychiatric hospital, the fact that the hospital serves both inpatient and outpatient means that more patients with mild conditions were recruited for this study. This needs to be considered when generalising the findings to more unwell patients.

The known-group validity assessment based on comparing the mean score in the composite A/D dimension and split subdimensions has shown a significant difference in mean scores for both composites and split dimensions with the clinical diagnosis or the severity level of anxiety or depression. Although both split subdimensions and the EQ-5D-5L A/D discriminated between groups with different levels of anxiety or depression, reported effect sizes differed across dimensions. Across anxiety severity groups, the largest effect sizes were found for the split subdimensions rather than the EQ-5D-5L A/D. Although EQ-5D-5L A/D had a better effect size than the anxiety subdimensions across depression severity groups, depression subdimensions had the largest effect sizes. The depression subdimension was more sensitive to anxiety and depression severity (with a large effect size). The split and composite measures revealed good known-group validity, with moderate to large effect sizes, especially when comparing groups with mild and severe conditions. However, no significant differences (with a small effect size) were found between groups with moderate and severe anxiety or moderately severe and severe depression, showing that disease severity might have influenced responses. The mean scores in the composite and split questions were able to discriminate the health state based on the severity from the less severe to the adjacent more severe state, which is consistent with the previous findings [23, 24] that indicated that the higher the score in condition-specific measures (GAD-7 and PHQ-9 scores), the lower the mean score value observed in composite and split questions. This finding might encourage using the EQ-5D as a health outcome measure in both clinical and community settings [25] to measure health outcomes, particularly for respondents with a mental health problem. The finding also provides evidence that the Amharic version of EQ-5D seems to be a valid and reliable outcome measure among respondents with major mental health disorders [17].

The Shannon indices were used to compare the degree of informativity of the two versions of the tool. The analysis showed that adding composite A/D dimension or split subdimensions to the tool's four dimensions increases absolute and relative informativity. Similarly, compared to standard EQ-5D-5L, adding either the two dimensions or one of the subdimensions increased both Shannon indices. This might indicate that the split versions have better informativity power than composite dimension. Despite this minor gain in informativity, the findings of this study demonstrated that EQ-5D-5L A/D has good known-group validity based on the types of diagnosis and severity of anxiety or depression. Therefore, prior to recommending splitting the composite A/D dimension, similar studies should be conducted in other patient groups and other non-English speaking countries to confirm our findings. Additionally, it is important to note that splitting the composite dimension will also have implications for valuation studies due to a larger descriptive system that may be associated with an increased cognitive burden [26]. Given that many valuation studies have been undertaken for the EQ-5D-5L to date, splitting the composite A/D dimension will also require new valuation studies. Therefore, before suggesting splitting the composite dimensions, more evidence from various demographic groups (e.g. in languages like Chinese or other mental health conditions, like bipolar disorder) and valuation studies should be gathered by splitting, dropping and adding EQ-5D-5L dimensions. Nevertheless, current findings demonstrate the need for careful interpretation of the descriptive system of EQ-5D in different populations [7].

Respondents’ comments regarding the reasons for their preference towards the composite or split questions reflected their understanding of the questions, relevance to their health conditions, and acceptability and perceived cognitive burden of the questions. This study has several strengths, including the use of validated Amharic tools to assess the self-reported severity of anxiety and depression and the inclusion of respondents with a diverse diagnosis of anxiety and/or depression. However, the interviewer-administered approach used to collect data might have introduced a social desirability bias, which could have influenced the response pattern seen, limiting the results' applicability to self-report contexts.

## Conclusions

Respondents with anxiety and depression are more likely to report problems on split subdimensions than the EQ-5D-5L A/D composite dimensions. Although both composite and split dimensions showed good known-group validity, the split ‘depression’ subdimension demonstrated better convergent validity with symptom severity scores. Overall, the findings suggest that the split subdimensions appear to perform slightly better than the composite EQ-5D-5L A/D dimension. However, more studies in other population groups (e.g. bipolar disorder) and countries (e.g. China) will be required to further confirm this conclusion.

## Supplementary Information

Below is the link to the electronic supplementary material.

Supplementary file1 (DOCX 41 KB)

## Data Availability

The data supporting the findings of this study are available within the article or its supplementary materials and further information can be accessed from the first author (YBB) upon request.
