# Supplementary material for: Examining the psychometric properties of a split version of the EQ-5D-5L anxiety/depression dimension in patients with anxiety and/or depression
Source: Qual Life Res. 2023 Feb 21;32(7):2025–36. doi: 10.1007/s11136-023-03372-7 (PMC10241678; doi:10.1007/s11136-023-03372-7)
Supplement: Supplementary file 1 — Supplementary file1 (DOCX 41 KB) [file 11136_2023_3372_MOESM1_ESM.docx]

# **Examining the psychometric properties of a split version of the EQ-5D-5L anxiety/depression dimension in patients with anxiety and/or depression**

**Authors:** Yared Belete Belay^1,2 *^, Cathrine Mihalopoulos^1^, Yong Yi Lee^1,3,4^, Brendan Mulhern^5^, Lidia Engel^1^

Email: [Yared.Belay1@monash.edu](mailto:Yared.Belay1@monash.edu); Telephone: +61 3 9903 0444

**Appendices**

Appendix 1 Survey content

| **Order** | **Instrument/question type** | **Language** | **Source** |
| --- | --- | --- | --- |
| 1 | Demographic information | Amharic | Patient |
| 2 | Interviewer administered EQ-5D-5L (no modification) | Amharic | Patient |
| 3* | Split anxiety and depression questions (modified from EQ-5D-5L composite anxiety/depression) | Amharic | Patient |
| 4 | Close-ended questions about which option respondents prefer as the best to express their health.   - The question that combines anxiety and/or depression - The question of split anxiety and depression subdimension | Amharic | Patient |
| 5 | Open-ended questions to get the respondents' feedback on why they prefer composite or split questions. | Amharic | Patient |
| 6 | EQ VAS (no modification) | Amharic | Patient |
| 7 | Patient health questionnaire-9 (PHQ-9) | Amharic | Patient |
| 8 | Generalised anxiety disorder 7-item (Gad-7) scale | Amharic | Patient |
| 9 | Checklist for clinical characteristics | English | Patient medical records |

* In half of the surveys, this question was placed between P/D and A/D questions of the standard EQ-5D-5L

Appendix 2 Distribution of responses across the EQ-5D-5L and the split anxiety and depression subdimensions (n=462)

Appendix 3 Chi-square test to show the difference in proportion of reported problems on the EQ-5D-5L composites and split domains

In total sample, the proportion of no problem report in composite and split questions was significantly different with (χ2(1, N = 924) = 40.55, p <0.001). These proportions were significantly different for respondents with anxiety (χ2(1, N = 298) = 14.34, p <0.001), depression (χ2(1, N = 318) = 25.75, p <0.001) or for respondents with both anxiety and depression (χ2(1, N =308) = 5.68, p = 0.017).

| Participants | EQ-Dimension | Sample size | Reported profile | | X^2^(1) | p-value |
| --- | --- | --- | --- | --- | --- | --- |
| Total sample (N=924) |  |  | No problems n (%) | Problems n (%) |  |  |
|  | EQ-5D-5L A/D | 462 | 141(0.31) | 321(0.69) | 40.55 | <0.001 |
|  | anxiety and depression subdimensions | 462 | 61 (0.13) | 401 (0.87) |  |  |
| Respondents with anxiety (N=298) | EQ-5D-5L A/D | 149 | 46 (0.31) | 103 (0.69) | 14.34 | <0.001 |
|  | anxiety and depression subdimensions | 149 | 19 (0.13) | 130 (0.87) |  |  |
| Respondents with depression (N=318) | EQ-5D-5L A/D | 159 | 83 (0.52) | 76 (0.48) | 25.75 | <0.001 |
|  | anxiety and depression subdimensions | 159 | 39 (0.25) | 120 (0.75) |  |  |
| Respondents with both anxiety and depression (N=308) | EQ-5D-5L A/D | 154 | 12 (0.08) | 142 (0.92) | 5.68 | 0.017 |
|  | anxiety and depression subdimensions | 154 | 3 (0.02) | 151 (0.98) |  |  |

Appendix 4 Summary of qualitative (content) analysis for the reasons why respondents preferred composite or split versions of the EQ-5D-5L

| Respondents who preferred the composite version of the questionnaire (N=163) | | | | | |
| --- | --- | --- | --- | --- | --- |
| No. | Category | Subcategory | n | % | Illustrative quote |
| 1 | Understanding of the questions | Clear to understand | 62 | 38% | "Both feelings are well represented in the combined question and are easily understandable. The question that combines anxiety and depression describes me well."  "It is easier to understand if the questions are combined and the more difficult it is for me to understand when they are split. It describes my health better. The combined question is short and clear for me." |
|  |  | Easy to respond to | 8 | 5% |  |
|  |  | Short | 5 | 3% |  |
|  |  | Describe adequately | 15 | 9% |  |
|  |  | Explain properly | 5 | 3% |  |
| 2 | Relevance to patients ‘health condition(s) | Describe their health conditions | 24 | 15% | "I have both feelings, and it is better to respond in a combined format."  "I feel depressed and anxious, so I like the question that combines anxiety and depression. The one that asked me about both anxiety and depression expressed all my feelings, so I chose them, and it is both short and precise." |
|  |  | Express the feeling | 1 | 1% |  |
| 3 | Acceptability and perceived cognitive burden of the questions | Difficult to differentiate the concept | 1 | <2% | "When you ask me about only depression or anxiety conditions, it makes me think much more about the difference between the two conditions and gets more difficult to answer; therefore, it is easier for me to respond when combined".  "When it is decomposed, it is a thought-provoking question; it takes time."  "The decomposed question bothered me a little bit and made me feel distressed."  Combined questions are easy to comprehend and respond to. |
|  |  | Avoids too much talking | 1 | <1% |  |
|  |  | Burden of thought | 1 | <1% |  |
| Respondents who preferred the split version of the questionnaire (N=299) | | | | | |
| No. | Category | Subcategory | n | % | Illustrative quote |
| 1 | Understanding of the questions | Easy to respond to | 15 | 5% | "The split questions are easier for me to describe my anxiety that is more relevant to me."  " Split questions are clearer and simple therefore I understand them easily and they also express how I feel today." |
|  |  | Clear to understand | 12 | 4% |  |
| 2 | Relevance to patients ‘health condition(s) | Describe their health conditions | 93 | 31% | "The split anxiety question tells a lot about my health and explains it very well, but depression is not my concern."  "When you ask me about depression, I feel better because it is the one that makes me feel better. It was simple to understand and was easy to respond to. I was surprised when you asked me together because depression is the one that affects me right now." |
|  |  | Feel comfortable | 9 | 3% |  |
|  |  | Easy to understand | 2 | <1% |  |
| 3 | Acceptability and perceived cognitive burden of the questions | Difficult to differentiate the concept | 2 | <1% | "There is no difference between the two situations; I could not tell the difference between the two diseases; why should I be concerned about the distinction between such similar diseases? Therefore, it would be better to ask me in combination."  The split question posed the burden of thinking long and hard about it |
|  |  | Distress related to questions | 1 | <1% |  |
|  |  | Burden of thought | 9 | 3% |  |

Note: The percentages shown in the table do not add up to 100% because some comments were deemed irrelevant and left out of the analysis

Appendix 5 Split EQ-5D-5L questions used for the survey

**English version**

EQ-5D-5L split subdimensions

**Next, I would like to ask you about ANXIETY. Would you say that:**

| 1. You are not anxious? |  |
| --- | --- |
| 1. You are slightly anxious? |  |
| 1. You are moderately anxious? |  |
| 1. You are severely anxious? |  |
| 1. You are extremely anxious? |  |

**Finally, I would like to ask you about DEPRESSION. Would you say that:**

| 1. You are not depressed? |  |
| --- | --- |
| 1. You are slightly depressed? |  |
| 1. You are moderately depressed? |  |
| 1. You are severely depressed? |  |
| 1. You are extremely depressed? |  |

Considering the questions about anxiety and/or depression above, which option do you think best expresses your health today? (*Note to interviewer*: please *remind them the questions*)

| 1. The question that combines anxiety and/or depression |  |
| --- | --- |
| 1. The question that split anxiety and/or depression |  |
|  |  |

Why?---------------------------------------------------------------------------------------------------------------------------------------------------------------------------------------------------------------------------------------------------------------------------------------------------------------------------------------------------------------------------------------------------------------------------------------------------------------------------------------------------------------------------------------------------
